# Italian consensus on the therapeutic management of uncomplicated acute hematogenous osteomyelitis in children

**DOI:** 10.1186/s13052-021-01130-4

**Published:** 2021-08-28

**Authors:** Andrzej Krzysztofiak, Elena Chiappini, Elisabetta Venturini, Livia Gargiullo, Marco Roversi, Carlotta Montagnani, Elena Bozzola, Sara Chiurchiu, Davide Vecchio, Elio Castagnola, Paolo Tomà, Gian Maria Rossolini, Renato Maria Toniolo, Susanna Esposito, Marco Cirillo, Fabio Cardinale, Andrea Novelli, Giovanni Beltrami, Claudia Tagliabue, Silvio Boero, Daniele Deriu, Sonia Bianchini, Annalisa Grandin, Samantha Bosis, Martina Ciarcià, Daniele Ciofi, Chiara Tersigni, Barbara Bortone, Giulia Trippella, Giangiacomo Nicolini, Andrea Lo Vecchio, Antonietta Giannattasio, Paola Musso, Elena Serrano, Paola Marchisio, Daniele Donà, Silvia Garazzino, Luca Pierantoni, Teresa Mazzone, Paola Bernaschi, Alessandra Ferrari, Guido Castelli Gattinara, Luisa Galli, Alberto Villani

**Affiliations:** 1 Paediatric and Infectious Disease Unit, Academic Department of Pediatrics, Bambino Gesù Children’s Hospital, IRCCS, Rome, Italy; 2 Paediatric Infectious Disease Unit, Anna Meyer Children’s University Hospital, Florence, Italy; 3 Department of Emergency, Acceptance and General Pediatrics, Bambino Gesù Children’s Hospital, IRCCS, Rome, Italy; 4 Rare Disease and Medical Genetics, Academic Department of Pediatrics, Bambino Gesù Children’s Hospital, Rome, Italy; 5 Infectious Disease Unit, IRCCS Istituto Giannina Gaslini, Genova, Italy; 6 Department of Imaging, Bambino Gesù Children’s Hospital, IRCCS, Rome, Italy; 7 Department of Experimental and Clinical Medicine, University of Florence, Florence, Italy; 8 Surgery Department, Traumatology Unit, Bambino Gesù Children’s Hospital, IRCCS, Rome, Italy; 9 Pediatric Clinic, Pietro Barilla Children’s Hospital, University of Parma, Parma, Italy; 10 Department of Pediatrics and Emergency, Pediatric Allergy and Pulmunology Unit, Azienda Ospedaliera-Universitaria “Consorziale-Policlinico”, Ospedale Pediatrico Giovanni XXIII, Bari, Italy; 11 Department of Health Sciences, Section of Clinical Pharmacology and Oncology, University of Florence, Florence, Italy; 12 Department of Orthopaedic Oncology and Reconstructive Surgery, AOU Careggi, Florence, Italy; 13 Pediatric Highly Intensive Care Unit, Fondazione Ca’ Granda Ospedale Maggiore Policlinico, IRCCS, Milan, Italy; 14 Department of Pediatric Orthopaedics, IRCCS Istituto ‘Giannina Gaslini’, Children’s Hospital, Genova, Italy; 15 Department of Pediatrics, ASST Santi Paolo e Carlo Hospital, Milan, Italy; 16 Pediatric Unit, San Martino Hospital, Belluno, Italy; 17 Section of Paediatrics, Department of Translational Medical Sciences, University of Naples Federico II, Naples, Italy; 18 Pediatric Emergecy Department, AORN Santobono-Pausilipon, Naples, Italy; 19 Division of Pediatric Infectious Diseases, Department for Woman and Child Health, University of Padua, Padua, Italy; 20 Pediatric Infectious Disease Unit, Regina Margherita Children’s Hospital, University of Turin, Turin, Italy; 21 Pediatric Emergency Unit, Policlinico di Sant’Orsola, Bologna, Italy; 22 Rome, Italy; 23 Microbiology Unit, Children’s Hospital Bambino Gesù, IRCCS, Rome, Italy; 24 Pediatric Unit, Cremona, Italy; 25 Child and Adolescent Health Institute, Bambino Gesù Children’s Hospital, IRCCS, Rome, Italy

**Keywords:** Paediatric infectious diseases, Paediatric osteomyelitis, Bone infections, Antibiotic therapy, Children, Paediatrics

## Abstract

**Background:**

Acute hematogenous osteomyelitis (AHOM) is an insidious infection of the bone that more frequently affects young males. The etiology, mainly bacterial, is often related to the patient’s age, but it is frequently missed, owing to the low sensitivity of microbiological cultures. Thus, the evaluation of inflammatory biomarkers and imaging usually guide the diagnosis and follow-up of the infection. The antibiotic treatment of uncomplicated AHOM, on the other hand, heavily relies upon the clinician experience, given the current lack of national guidelines for the management of this infection.

**Methods:**

A systematic review of the studies on the empirical treatment of uncomplicated AHOM in children published in English or Italian between January 1, 2009, and March 31, 2020, indexed on Pubmed or Embase search engines, was carried out. All guidelines and studies reporting on non-bacterial or complicated or post-traumatic osteomyelitis affecting newborns or children older than 18 years or with comorbidities were excluded from the review. All other works were included in this study.

**Results:**

Out of 4576 articles, 53 were included in the study. Data on different topics was gathered and outlined: bone penetration of antibiotics; choice of intravenous antibiotic therapy according to the isolated or suspected pathogen; choice of oral antibiotic therapy; length of treatment and switch to oral therapy; surgical treatment.

**Conclusions:**

The therapeutic management of osteomyelitis is still object of controversy. This study reports the first Italian consensus on the management of uncomplicated AHOM in children of pediatric osteomyelitis, based on expert opinions and a vast literature review.

**Supplementary Information:**

The online version contains supplementary material available at 10.1186/s13052-021-01130-4.

## Background

Osteomyelitis (OM) is an acute or chronic infection of the bone that more frequently affects preschool children, with a male-to-female ratio of 2:1. Long bones and vertebrae are the most freque ntly affected skeletal segments [1–5]. The etiology of the infection is bacterial in most cases [1]. The most common type of bone infection in children is Acute Hematogenic Osteomyelitis (AHOM) [5], whose pathogens may diverge based on children’s age (Table 1).

**Table 1:** Age distribution of most frequently involved pathogens in pediatric AHOM

Age	Pathogens
**< 3 months**	*S. aureus* *E. coli* *H. influenzae* *N. gonorrhoeae* (in case of congenital infection) *Streptococcus β haemolyticus group B* *C. albicans*
**3 months – 5 years**	*S. aureus* *K. kingae* *Streptococcus β haemolyticus group A* *S. pneumoniae* (especially under 2 years-old) *H. influenzae* type B (rare in fully vaccinated immunocompetent patients)
**> 5 years**	*S. aureus* *Streptococcous β haemolyticus group A* *N. gonorrhoeae* (in sexually active adolescents)

Clinical presentation of AHOM is highly variable and depends on multiple factors, including age, causative organism, anatomical site, and presence of an underlying disease [3, 4]. Symptoms’ onset is often insidious, especially in newborns and younger patients [6, 7]. Moreover, while the lower extremities are more frequently affected compared to the upper ones, the involvement of a single bone segment is much more frequent than multifocal infections [2, 3].

The AHOM diagnostic work-up includes the evaluation of several inflammatory markers such as white blood cell count, erythrocyte sedimentation rate, and C-reactive protein level. The combination of those biomarkers can be helpful for AHOM diagnosis as well as for the evaluation of treatment response and monitoring, while procalcitonin is less useful due to its low sensitivity and high cost [1–3, 8, 9].

Overall, microbial cultures show lower capability to diagnose AHOM depending on patient age, location of the infection, techniques used, as well as laboratory experience. Since the hematogenous dissemination during AHOM has been documented as a common occurrence in children, blood cultures represent a valuable diagnostic tool to establish the etiology, even if in a high percentage of children with AHOM (30–50%), cultures do not allow the isolation of the germ responsible for the infection [1–4, 10]. On the other hand, the microbial culture of the material derived from the infected bone constitutes the diagnostic gold standard [1, 3]. In recent years, the introduction of new diagnostic techniques, i.e. molecular tests on bone biopsy, and/or mass spectrometry with MALDI-TOF increased the overall diagnostic performance and ensured a rapid identification of the pathogen [1].

The initial diagnostic approach when AHOM is suspected is the imaging because of its role in excluding other possible differential diagnoses, such as traumatic or non-infectious lesions. X-rays own a reasonably high specificity (75–83%), while its sensitivity remains lower (43–75%) than other techniques [11]. Magnetic Resonance Imaging (MRI) is the gold standard in the diagnosis of osteomyelitis, due to its higher sensitivity and specificity (respectively of 82–100% and 75–99%) [12]. MRI is also able to document bone edema, the first non-specific sign of osteomyelitis, within 24–48 h from the infection onset. MRI is also increasingly used in evaluating multifocality [13], and it has almost completely replaced bone scintigraphy, being more sensitive and avoiding exposure to radiations. Ultrasound instead is useful in identifying joint effusion, soft tissue abscess, and sub-periosteal collection, findings that may be associated with AHOM [11].

## Methods

A systematic literature review on the empirical treatment of uncomplicated AHOM in children was carried out in order to evaluate the available data from the studies published between 2009 and March 2021.

A multidisciplinary Italian panel of experts was set up to address the following scientific issues:

- antibiotic molecules for intravenous (iv) empiric therapy
- duration of intravenous antibiotic therapy
- factors influencing the switch from iv therapy to oral (os) therapy
- duration of os therapy
- total duration of antibiotic treatment

The characteristics and results of the selected studies are summarized in Supplementary Table 1, and the panel analyzed the results through frontal and online discussions. Consent was obtained by using the Delphi method.

All articles in English and Italian published from January 1, 2009, to March 31, 2021, concerning the empirical antibiotic therapy of uncomplicated AHOMs in children aged between 28 days and 18 years were selected using Pubmed and Embase search engines.

The search was conducted exploiting the two strings shown below:

### Pubmed search string

((((osteomyelitis) OR ((bone* OR osteoarticular OR musculoskeletal) AND infection*)) AND (manag* OR therap* OR antibiotic* OR treatment*)) AND (child* OR pediatric* OR paediatric* OR kid* OR infant))

### Embase search string

(((‘osteomyelitis’/exp. OR osteomyelitis:ti,ab) AND acute:ti,ab) OR ((bone:ti,ab OR osteoarticular:ti,ab OR musculoskeletal:ti,ab) AND infection:ti,ab)) AND (manag*:ti,ab OR therap*:ti,ab OR antibiotic*:ti,ab OR treatment*:ti,ab) AND ([infant]/lim OR [child]/lim OR [preschool]/lim OR [school]/lim OR [adolescent]/lim) AND [2009–2020]/py AND ([english]/lim OR [italian]/lim)

Criteria used to select articles are shown in Table 2.

**Table 2:** Inclusion and exclusion criteria of selected studies

Inclusion criteria	Exclusion criteria
Subacute or acute infectious osteomyelitis due to bacterial etiology	Subacute or chronic non-infectious osteomyelitis or articles related to non-bacterial (e.g., fungal, or mycobacterial) osteomyelitis
Osteomyelitis in children aged 28 days to 18 years-old	Osteomyelitis in patients aged < 28 days and > 18 years-old
Uncomplicated osteomyelitis	Complicated osteomyelitis
Osteomyelitis not caused by surgery or trauma	Osteomyelitis caused by surgery or trauma
Osteomyelitis onset in healthy children	Osteomyelitis in children with underlying chronic, onco-hematological or immunodeficiency disorders
Cohort studies or case reports including more of 10 patients	Guidelines

## Results

**Fig. 1:**
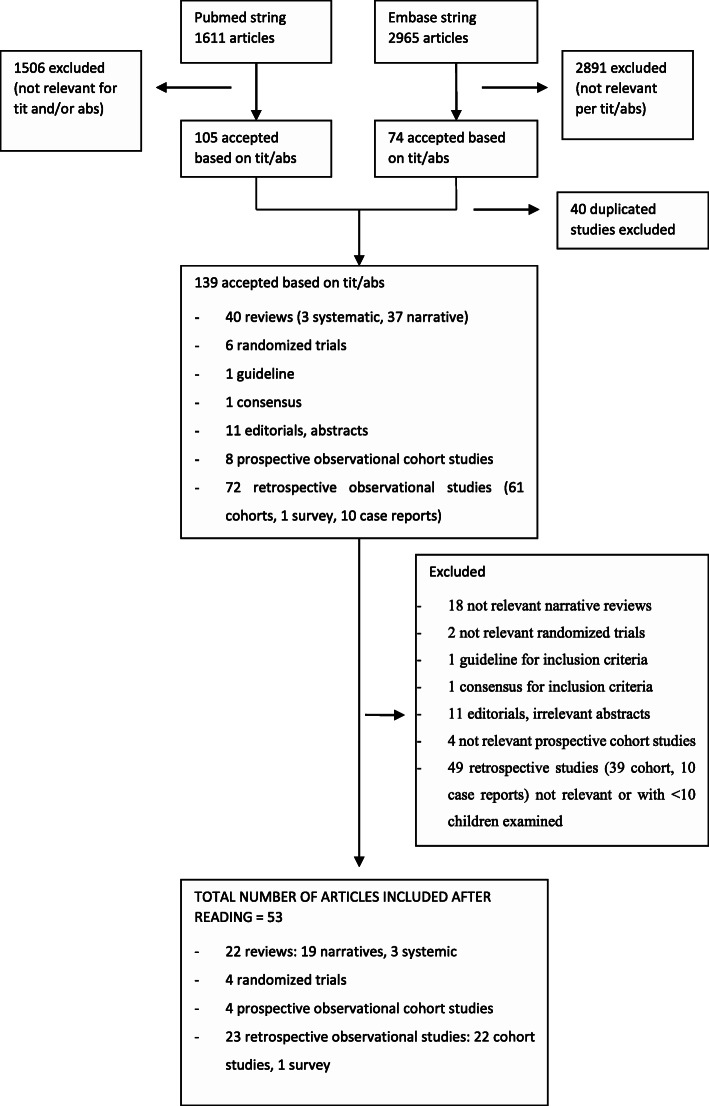
Articles selection’s search tree algorithm

Out of 4576 articles, 139 were selected based on title and abstract and 53 were considered relevant as they satisfied the established inclusion/exclusion criteria (Fig. 1).

### Bone penetration of antibiotics

The bone penetration of the different classes of antibiotics was evaluated by pharmacokinetic studies using different methodologies [14–16]. According to the available data, bone penetration of the main antibiotics used for treating AHOM is reported in Table 3.

**Table 3:** Percentage of bone penetration of the main antibiotics used in AHOM

Antibiotic	Percentage of bone penetration		
	Boselli 1999 [14]	Landrsdorfer 2009 [15]	Thabit 2019 [16]
BETA-LACTAMS			
**Amoxicillin**	17–31%	18–20%	10% (amoxi-clavulanate)
**Clavulanic**	–	10–15%	–
**Ampicillin**	16%	11–71%	–
**Sulbactam**	–	17–71%	–
**Piperacillin**	18–23%	18–23% or 15%	15% (piperacillin-tazobactam)
**Tazobactam**	22–26%	22–26%	–
**Flucloxacillin**	8–15%	5–15%	65%
**Oxacillin**	–	11%	21%
CARBAPENEMS			
**Ertapenem**	–	10–20%	35%
**Meropenem**	–	–	50%
CEPHALOSPORINS			
**Ceftriaxone**	–	7–17%	–
**Cefazolin**	18%	18%	25%
**Cefepime**	–	46–76%	–
**Cefuroxime**	14–23%	–	–
**Cefotaxime**	8,8%	–	–
**Ceftazidime**	20–35%	54%	49%
MACROLIDES			
**Erythromycin**	28.5–39%	18–28%	–
**Azithromycin**	–	250–630%	–
GLYCOPEPTIDES			
**Vancomycin**	60.8%	5–67%	20–40%
**Teicoplanin**	14–290%	50–64%	–
AMINOGLYCOSIDES			
**Gentamicin**	14–55%	16–33%	–
**Amikacin**	15–30%	–	–
OTHERS			
**Metronidazole**	–	–	50%
**Linezolid**^a^	–	23–51%	44%
**Daptomycin**	–	12–55% or 108%	20%
**TMP-SMX**	11–60%	15–50%	25%
**Rifampicin**	17–41%	20–25%	40%
**Tigecycline**^a^	–	35–195% or 47%	–
**Clindamycin**	98.3%	21–45%	26%

^a^Not registered for pediatric use

However, it should be considered that the minimum inhibitory concentration (MIC) of the isolated bacteria [14] is also determinant for the choice of the best antibiotic.

### Intravenous antibiotic therapy

The scientific literature review revealed heterogeneous data on the empiric antibiotic therapy of AHOM.

The most commonly administered antibiotics are anti-staphylococcal penicillins (oxacillin, nafcillin, cloxacillin, and flucloxacillin) and cephalosporins. Among the latter, the most commonly used first generation molecules are cefazolin (the only one available in Italy) [1–4, 17–26], cephalothin [4, 20, 21, 23], and cefradine [20, 21, 23], while the most used second generation one is cefuroxime [18, 22]. Third generation cephalosporins such as ceftriaxone and cefotaxime [3, 27] are less frequently used.

The antibiotic choice should consider several factors, including age, drug toxicity, bone penetration, and local prevalence of methicillin-resistant *S. aureus* (MRSA) and Extended-Spectrum Beta-Lactamases (ESBL) bacteria. According to age and etiology, intravenous treatment options for uncomplicated AHOM are shown in Tables 4 and 5.

**Table 4:** Intravenous treatment of non-complicated AHOM according to age

Age	Empiric treatment (I choice)	Empiric treatment (II choice)
**< 3 months**	Ampicillin-sulbactam OR Cephazolin + Gentamycin	Oxacillin + Gentamycin OR Amoxicillin/clavulanate + Gentamycin OR Cefotaxime + Oxacillin (if low prevalence of ESBL)
**3 months- 5 years**	Cephazolin	Amoxicillin/clavulanate OR Ampicillin/sulbactam OR Ceftriaxone + Clindamycin or Glycopeptides (if MRSA prevalence > 10%)
**> 5 years**	Oxacillin OR Cephazolin OR Clindamycin	Amoxicillin/clavulanate OR Ampicillin/sulbactam OR Ceftriaxone OR Ceftazidime + Clindamycin or Glycopeptides (if MRSA prevalence > 10%)

**Table 5:** Intravenous antibiotic dosage

Antibiotic	Recommended dose
**Amoxicillin/clavulanate**	75–100 mg/kg daily of amoxicillin in 3–4 divided doses (max 1 g/dose)
**Ampicillin/sulbactam**	100–200 mg/kg daily of ampicillin in 4 divided doses (max 2 g/dose)
**Cephazolin**	150 mg/kg daily in 3–4 divided doses (max 2 g/dose)
**Ceftazidime**	150 mg/kg daily in 3 divided doses (max 2 g/dose)
**Ceftriaxone**	50–100 mg/kg daily (max 2 g)
**Clindamycin**	45 mg/kg daily in 3 divided doses (max 900 mg/dose)
**Oxacillin**	150–200 mg/kg daily in 4 divided doses (max 2 g/dose)
**Gentamycin**	neonates ≥35 weeks of gestational age: 4 mg/kg daily during the first week of life, then 5 mg/kg daily > 1 month-10 years: 8 mg/kg the first day, then 6 mg/kg daily > 10 years: 7 mg/kg daily the first day, then 5 mg/kg daily
**Linezolid**^a^	< 12 years: 30 mg/kg daily in 3 divided doses (max 600 mg/dose) > 12 years: 600 mg twice a day
**Vancomycin**	45 mg/kg daily in 3 divided doses

^a^Not registered for pediatric use

### Methicillin-resistant *S. aureus*

In western countries, the overall prevalence of community-acquired MRSA has increased in recent years and, more specifically, Italian data show high but stable rates of oxacillin-resistant strains [28]. Thus, knowledge of local epidemiology is crucial in establishing the empirical therapy of AHOM. In this regard, data are often little or difficult to analyze because usually not homogeneous and frequently not discriminating between adults and children [5, 7, 29].

There is a broad debate on the empirical use of antibiotics active against MRSA. In fact, according to the European Society of Pediatric Infectious Diseases (ESPID) 2017 guidelines, such drugs should be reserved for areas with MRSA prevalence rates > 10% [7].

In cases of strong clinical suspicion of MRSA osteomyelitis or areas where the local prevalence of MRSA is greater than 10%, the drugs of choice are clindamycin, vancomycin, and linezolid [1–4, 17, 25, 26, 30–32], bearing in mind that the use of the linezolid is off-label in the pediatric age; the use of daptomycin is indicated in case of first-line therapeutic failure [33].

### Panton-valentine Leukocidin–producing *S. aureus*

In recent years, the pathogenic role of Panton-Valentine Leukocidin-producing *S. aureus* (PVL-SA) has been highlighted, with a reported prevalence in Italy up to 10% of all pediatric AHOM cases [34]. PVL is a toxin causing leukocyte lysis by forming pores in the membrane, with the consequent risk of severe lung, bone, skin, and soft tissues infections [35].

Antibiotic therapy of PVL-SA must aim at inhibiting toxin production: thus, antibiotics inhibiting protein synthesis such as clindamycin, linezolid, or rifampicin are indicated [35].

However, for uncomplicated pediatric AHOM, indications on empirical administration of anti-PVL antibiotics are currently lacking [2, 7].

### *K. kingae*

*K. kingae* is among the most frequently isolated pathogens in AHOM of children between 3 months and five years of age [1, 2, 7]. A few studies show age stratification of empirically used antibiotics [1, 2]. In these studies, the empirical use of a cephalosporin, or ampicillin/ampicillin-sulbactam, is suggested in this age group to ensure coverage of *K. kingae*, as anti-staphylococcal penicillins, clindamycin, and glycopeptides are ineffective against this pathogen [36].

## Oral antibiotic therapy

In most studies, oral therapy of AHOM is done with high-dose cephalosporin, clindamycin, or amoxicillin-clavulanic acid, alone or in combination with rifampicin [1–5, 10, 19, 21–24, 37–41].

Cephalexin is the oral drug of choice after parenteral therapy with first-generation cephalosporins, as its action profile against penicillin-resistant *S.aureus* and *S.pyogenes* is adequate in vitro, with a good absorption and tolerance profile in pediatric patients [19–24].

Clindamycin is a safe, inexpensive, and effective against MSSA and MRSA, available both for intravenous and oral administration [23]. The clinical and bacteriological response to clindamycin is generally excellent when the pathogen is susceptible, with optimal serum and tissue concentrations [19–24]. Clindamycin may therefore be a valid choice when the *S.aureus* strain is fully susceptible.

According to the literature, amoxicillin-clavulanate is the most widely used antibiotic in European observational studies, either as monotherapy or combined with rifampicin, in settings with a low prevalence of MRSA [10, 40]. In a recent survey on empirical oral therapy of AHOM sent to 31 Italian pediatric centers, amoxicillin-clavulanate was found to be the first choice in all age groups [42]. However, only limited data are available in the literature regarding its effectiveness, and this regimen is associated with a higher rate of side effects when compared with narrower spectrum molecules [43–45]. Nonetheless, the ESPID guidelines emphasize that these side effects are usually non-severe and transient [7].

Trimethoprim/sulfamethoxazole could be used to manage osteomyelitis, even as monotherapy, when the clinical condition is stable [10, 21, 38, 39, 46–49]. This drug represents an attractive option due to its anti-MRSA activity: time-kill kinetic studies have demonstrated bactericidal action at concentrations four times higher than the MIC. Its bone penetration profile is satisfactory (approximately 50% of serum levels for trimethoprim and 15% for sulfamethoxazole). Both oral and parenteral formulations are available. However, a recent study suggested limiting the use of trimethoprim/sulfamethoxazole to severe cases, especially when associated with bacteremia [50].

Rifampicin may also play a role in the combination therapy for *S.aureus* driven bone infections. Both oral and intravenous formulations are available, with excellent oral bioavailability. Its efficacy is still a matter of debate, and much of its use is based on clinical practice, above all for orthopedic prostheses-related staphylococcal infections [37, 51].

In clindamycin-resistant MRSA infections, linezolid may play an important role. It is considered by many authors as an alternative in MRSA bone and joint infections, first intravenous and then as an oral therapy, particularly in patients with systemic reactions to intravenous vancomycin [52–54], such as the red-man syndrome. It is a good option for managing serious infections that may require long-term therapy, including osteomyelitis, due to its excellent bone penetration. It has an oral bioavailability of 99–100%, so it can easily be switched from parenteral to oral treatment. Neuropathic signs are described among irreversible severe adverse events, especially after prolonged courses of therapy in adults [55]. At present this drug is not registered by European Medicine Agency for pediatric use, even if US-Food and Drug Administration has approved linezolid for several infections in pediatrics.

According to age group, oral treatment options for the therapy of uncomplicated AHOM according to etiology are suggested and shown in Tables 6 and 7.

**Table 6:** Suggested oral therapy in uncomplicated AHOM by age group

Age	Oral therapy
**Unknown aetiological agent (age < 5 years)**^a^	Cephalexin OR Amoxicillin-clavulanate +/− Rifampicin
**Unknown aetiological agent (age > 5 years)**	Cephalexin OR Flucloxacillin OR Clindamycin
***S. aureus***	Cephalexin OR Flucloxacillin OR Clindamycin^b^ OR TMX-SMX + Rifampicin^b^ OR Linezolid^bc^
***K. kingae***	Amoxicillin-clavulanate OR Cefixime OR Cefpodoxime OR Cefazolin OR Trimethoprim/sulphamethoxazole
***S. pyogenes***	Cephalexin OR Flucloxacillin OR Amoxicillin

^a^Oral antibiotic therapy is not indicated in infants < 3 months old

^b^If MRSA or PVL-SA is suspected or confirmed

^c^Not registered for pediatric use

**Table 7:** Oral antibiotic dosage

Antibiotic	Recommended dose
Cephalexin	100 mg/kg daily in 4 divided doses (max daily dosage 4 g)
Amoxicillin-clavulanate	80 mg/kg daily in 3 divided doses (max daily dosage 2 g)
Amoxicillin	75–100 mg/kg daily in 3 divided doses (max daily dosage 3 g)
Clindamycin	30–40 mg/kg daily in 3–4 divided doses (max daily dosage 1.8 g)
TMP-SMX	8 mg/kg daily of TMP in 2 divided doses (max daily dosage 320 mg of TMP)
Rifampicin	10–20 mg/kg daily in 1–2 divided doses (max daily dosage 600 mg)

## Length of treatment and switch to oral therapy in AHOM

The total duration (intravenous and oral) of AHOM therapy is widely debated in the scientific literature. The mean total length of treatment of uncomplicated AHOM is approximately four weeks, ranging from 3 to 6 weeks [1–5, 19, 21–26, 46, 47, 56].

Previously, children with osteomyelitis were switched to oral therapy after several weeks of iv treatment (usually 2–4 weeks) and often close to recovery [5]. However, prolonged intravenous antibiotic treatment is associated with longer hospitalization, higher costs, and a central venous catheter placement, with the risk of mechanical complications (i.e., occlusion, rupture, dislocation), venous thrombosis, and catheter-related infection.

In the last decade, several studies were conducted to evaluate the possibility of an early switch to oral therapy (within 2–7 days of starting iv treatment) [21–26, 40, 57, 58]. No difference in terms of treatment failure was observed. However, these studies were conducted in settings with low MRSA prevalence.

The leading indicators for switching from intravenous to oral therapy are still debated in the literature [1–5, 21–25, 31, 59]. In more detail, to guide the switch from intravenous antibiotics to oral therapy, several qualitative and quantitative variables need to be verified, such as good clinical status, improvement of local signs, apyrexia for more than 48 h, and reduction of C-Reactive Protein (CRP) values by at least 50% (< 2–3 mg/dl) [22, 58].

The 2017 ESPID Guidelines recommend to switch to oral therapy after 2–4 days of intravenous antibiotics when the patient shows:

- clinical improvement (afebrile or decreasing body temperature for 24–48 h);
- improvement of local symptoms;
- lack of signs related to complication;
- 30–50% decrease of CRP (compared to the peak value in the course of the infection);
- negative culture tests:
- absence of pathogens such as MRSA or PVL-SA, or other antibiotic-resistant pathogens [7].

The duration of oral therapy in uncomplicated AHOM is usually 3–4 weeks, with close monitoring of clinical manifestations, inflammatory markers, and drug tolerability [1, 2, 22].

There is insufficient data to support short iv therapy with a subsequent switch to oral treatment in infants under 3 months of age. According to some studies, infants should receive no less than 4 weeks of parenteral antibiotic therapy exclusively [6, 31]. Switching to oral therapy can only be considered in infants without severe complications who are able to take oral medications.

Suggestions for switching to oral therapy according to the intravenous treatment previously performed are given in Table 8.

**Table 8:** Proposed switch to oral therapy on the basis of intravenous therapy

Intravenous therapy	Proposed oral therapy
Cefazolin	Cephalexin
Amoxicillin-clavulanate	Amoxicillin-clavulanate
Ampicillin	Amoxicillin
Ampicillin-sulbactam	Amoxicillin-clavulanate
Oxacillin	Flucloxacillin OR Cephalexin^a^
Clindamycin	Clindamycin OR Trimethoprim/sulfamethoxazole^a^
Ceftriaxone	Amoxicillin-clavulanate
Ceftriaxone + Clindamycin or glycopeptides	Trimethoprim/sulfamethoxazole + Rifampicin
Vancomycin	Trimethoprim/sulfamethoxazole + Rifampicin OR Linezolid^b^

^a^If ingestion of tablets is difficult/compromised

^b^Not registered for pediatric use

## Surgical treatment

According to the latest ESPID Guidelines, the approach to osteoarticular infections must include, when possible, the drainage of purulent material and the collection for culture samples, in order to isolate the infectious agent and verify its antimicrobial susceptibility [7].

Nonetheless, the most common approach in treating AHOM is medical therapy [1], since conservative intervention is effective in 90% of cases [5]. Surgery is reserved for those cases where antibiotic therapy alone is insufficient for clinical and laboratory improvement.

## Discussion and suggestions for recommendations

The present review on uncomplicated AHOM in children shows that data regarding the epidemiology as well as the type and duration of antibiotic therapy are discordant and not homogeneous.

Thus, the recommendations that the Italian panel suggest for empirical therapy in uncomplicated AHOMs in children between 28 days and 18 years are the following:

- infants < 3 months of age: initial empiric use of ampicillin/sulbactam + gentamicin or cefazolin + gentamicin;
- if the prevalence of MRSA is less than 10%
  - Infants and children aged 3 months to 5 years: initial empiric use of a first- or second-generation iv cephalosporin;
  - Children > 5 years of age: initial empiric use of an iv anti-staphylococcal penicillin or a first- or second-generation cephalosporin or clindamycin, if the prevalence of MRSA is less than 10%;
- in case of therapeutic failure demonstrated by clinical and/or laboratory data, switch to second-line therapy (see Table 4);
- in infants and children > 3 months of age, switch to oral therapy within 5–7 days of iv therapy, after verifying the compliance of the child and the family;
- when switching from iv to oral therapy, prioritize the use of cephalexin or amoxicillin-clavulanic acid, possibly associated with rifampicin; among the anti-staphylococcal penicillin, although difficult to use due to the type of formulation that reduces its compliance, favor the use of flucloxacillin, well tolerated and with good bone penetration;
- monitor clinical signs and inflammatory biomarkers 48 to 72 h after the start of iv therapy and before switching to oral therapy (avoid switching in case of worsening of clinical conditions or increase of inflammatory biomarkers);
- in case of clinical worsening, modify iv therapy to ensure adequate coverage against resistant pathogens;
- favor the use of rifampicin and trimethoprim-sulfamethoxazole, given the good bone penetration and the optimal cost/benefit ratio; administer rifampicin in 2 daily doses, always in combination with other antibiotics, in order to avoid development of resistant strains;
- clindamycin should be used with caution due to the high prevalence of resistance in Italy (> 25%), the type of capsule formulation, and frequent gastrointestinal side effects that may reduce compliance with treatment;
- discontinue oral therapy 3 to 5 weeks after the switch if there are no complications;
- establish close clinical, laboratory, and instrumental follow-up during the administration of oral therapy and in the weeks following the complete discontinuation of therapy, possibly by a multidisciplinary team including pediatricians, an infectious disease specialist, and an orthopedics.

## Supplementary Information

**Additional file 1.**

## Data Availability

Not applicable.

## Abbreviations

AHOM: Acute Hematogenic Osteomyelitis
IV: Intravenous
MALDI-TOF: Matrix Assisted Laser Desorption Ionization – Time of Flight
MIC: Minimum Inhibitory Concentration
MRI: Magnetic Resonance Imaging
MRSA: Methicillin-resistant *S. aureus*
OM: Osteomyelitis
PVL-SA: Panton-Valentine Leukocidin-producing *S. aureus.*
