# Supplementary material for: Italian consensus on the therapeutic management of uncomplicated acute hematogenous osteomyelitis in children
Source: Ital J Pediatr. 2021 Aug 28;47:179. doi: 10.1186/s13052-021-01130-4 (PMC8403408; doi:10.1186/s13052-021-01130-4)
Supplement: Supplementary file 1 — Additional file 1. [file 13052_2021_1130_MOESM1_ESM.docx]

**Supplementary Table 1.**

| Autore, anno, luogo | Tipo di studio | Popolazione | Metodo | Risultati | Fallimenti/ conclusioni |
| --- | --- | --- | --- | --- | --- |
| Pääkkönen M  2015  Finlandia | Trial randomizzato | 11 | due gruppi: durata totale della terapia di 20 giorni (n=5), durata totale della terapia di 30 giorni (n=6)  i pazienti sono stati trattati con clindamicina (40mg/kg/die, qid)(2 short+4 long)o con cefalosporina di prima generazione (150 mg/kg/die, qid) (3 short+2 long) | non ci sono state differenze nei due gruppi di studio (short e long therapy) né sul tipo di antibiotico | 0 |
| Pääkkönen M  2013  Finlandia | Trial randomizzato | 345 | i pazienti sono stati randomizzati in due gruppi: 77% (n=265)coltura-positiva (usato come controllo), 23% (n=80)coltura negativa  i pazienti sono stati randomizzati in due gruppi in base alla durata totale della terapia: breve durata (20 giorni se con diagnosi di osteomielite, 10 giorni se con diagnosi di artrite settica), lunga durata( 30 giorni se osteomielite e 30 giorni se artrite settica)  la durata media della terapia intravenosa è stata di 3 giorni per entrambi i gruppi  nel gruppo coltura-negativa 48 pazienti sono stati trattati con clindamicina (40mg/kg/die, qid), 29 con cefalosporina di prima generazione (150 mg/kg/die, qid), 3 pazienti con una terapia antibiotica combinata, ampicillina o amoxicillina data come adiuvante in 8 pz  nel gruppo coltura-positivo è stata somministrata a 70 pazienti una cefalosporina di prima generazione, a 99 pazienti clindamicina, ampicillina, o amoxicillina, a 24 pazienti ampicillina o amoxicillina, a 72 pazienti una terapia antibiotica combinata | non ci sono state differenze nei due gruppi coltura-positiva e coltura-negativa | 3 pazienti coltura-positiva (1%) e 1 paziente con coltura negativa (1%) hanno sviluppato sintomi a 1 anno dalla dimissione |
| Peltola H  2010  Finlandia | Trial randomizzato | 131 | i pazienti sono stati randomizzati in due gruppi: terapia durata breve 20 giorni (n=67) e terapia durata lunga 30 giorni (n=64)  la durata media della terapia endovenosa è stata 3,7 giorni nel gruppo durata breve, 4,1 giorni nel gruppo lunga durata  è stata somministrata la clindamicina (40mg/kg/die qid) da sola in 75 pazienti, in associazione ad amoxicillina in 5 pazienti, una cefalosporina di prima generazione (cefradina o cefalexina o cefadrossile 150 mg/kg/die, qid) da sola in 37 pazienti, combinata con amoxicillina in 5 pazienti  in bambini tra gli 0 e 4 anni somministrata ampicillina o amoxicillina (200 mg/kg/die, qid) fino al 1997 per alto rischio di Heamophilus influenzae in epoca pre-vaccino | la maggior parte dei pazienti sono guariti rapidamente, senza differenze tra i due gruppi | 126 pazienti in follow-up: 2 sono risultati con sequele minori (uno per gruppo) a 12 mesi |
| Peltola H  2011  Finlandia | Trial randomizzato | 252 (dopo criteri di esclusione 169) | i pazienti sono stati randomizzati in due gruppi: terapia durata breve (20 giorni) e terapia durata lunga (30 giorni)  la durata media della terapia endovenosa è stata 3-4 giorni  i pazienti sono stati ulteriormente randomizzati: è stata somministrata clindamicina (40mg/kg/die qid) in 99 pazienti per un totale di 23 giorni, cefalosporina di prima generazione (cefradina o cefalexina o cefadrossile 150 mg/kg/die, qid)in 70 pazienti per un totale di 24 giorni  6 pazienti del gruppo della clindamicina e 8 del gruppo della cefalosporina hanno ricevuto per i primi giorni ampicillina (200 mg/kg/die, qid) | 99 su 99 pazienti trattati con clindamicina e 69 su 70 pazienti trattati con cefalosporine sono guariti con il primo trattamento  non ci sono state differenze statisticamente significative osservate nei due gruppi | 2 pazienti con sequele minori nel follow-up, uno per ciascun gruppo |
| Islam S  2019  USA | Osservazionale  retrospettivo  coorte | 74 | il periodo in studio è stato diviso in due gruppi- anno:  time1 dal 2013 al 2014  time2 dal 2015 al 2016  74% (n=55) coltura-positiva: 65% (n=36) MSSA; 20% (n=11) MRSA  Gruppo time1:  infezioni osteoarticolari n=31  durata terapia IV ≤ 7 giorni: 37%  uso del PICC: 65%  osteomieliti n=28  durata mediana della terapia IV in pazienti con osteomieliti 17 giorni  uso del PICC nei pazienti con osteomieliti 68%  PICC-casi MRSA 100%(n=5)  PICC- casi MSSA 71%(n=14)  PICC- casi non Staphylococcus aureus 44% (n=9)  Gruppo time2:  infezioni osteoarticolari n=43  durata terapia IV ≤ 7 giorni: 65%  uso del PICC: 21%  osteomieliti n=28  durata mediana della terapia IV in pazienti con osteomieliti 7 giorni  uso del PICC nei pazienti con osteomieliti 29%  PICC-casi MRSA 25%(n=4)  PICC-casi MSSA 31% (n=16)  PICC- casi non Staphylococcs aureus 25% (n=8) | Gruppo time1:  visita al pronto soccorso per cause relative al PICC 16%(n=5)  visita il pronto soccorso per cause relative all'infezione osteoarticolare 13%(n=4)  visita il pronto soccorso per altre cause 16% (n=5)  visita il pronto soccorso per cause correlate al PICC o all'infezione osteoarticolare 29% (n=9)  riammissione per PICC o infezioni osteoarticolari 26%(n=8)  Gruppo time2:  visita al pronto soccorso per cause relative al PICC 0%(n=0)  visita il pronto soccorso per cause relative all'infezione osteoarticolare 0%(n=0)  visita il pronto soccorso per altre cause 16%(n=7)  visita il pronto soccorso per cause correlate al PICC o all'infezione osteoarticolare 0% (n=0)  riammissione per PICC o infezioni osteoarticolari 0%(n=0) | le prove a supporto del passaggio precoce alla terapia orale per i bambini affetti da infezioni osteoarticolari è consistente e robusta |
| Kargel JS, Sammer DM, Pezeshk RA, Cheng J. ;  2018  USA | Osservazionale  retrospettivo  coorte | 21 | analisi retrospettiva in pazienti pediatrici con diagnosi di osteomielite della mano trattati con terapia orale | trattamento iniziale:  1. antibiotico terapia orale (n=17)  -successo del trattamento PO 13/17  -passaggio alla terapia IV 1/17  -passaggio al debridement chirurgico 3/17  2. Debridement chirurgico (n=4)  -successo della terapia antibiotica orale post-chirurgica 4/4  -passaggio alla terapia IV 0/4 | molti casi di osteomieliti della mano nei bambini possono essere trattati con una terapia antibiotica orale prolungata di 6-8 settimane  l'antibiotico più utilizzato è stato la clindamicina |
| Alcobendas R, Remesal A, Murias S, Nuñez E, Calvo C.;  2018  Spagna | Osservazionale prospettico coorte | 253 | 25 pazienti trattati ambulatorialmente con la sola terapia orale  criteri di selezione:  - buone condizioni generali di salute  - precedente artrocentesi se pazienti con artrite settica  -precedenti campioni ematici e del liquido sinoviale per esami colturali e PCR per Kingella kingae  - possibilità di uno stretto follow-up ambulatoriale  - tolleranza orale alla terapia  228 pazienti ospedalizzati trattati con la terapia endovenosa seguita dalla terapia orale | 1. gruppo pazienti trattati con sola terapia orale  - completa risoluzione nel 100% dei casi  - non ci sono state complicazioni  2. gruppo pazienti trattati con terapia endovenosa e orale:  - ci sono state complicazioni nel 24% dei casi  - ci sono state sequele nel follow-up nel 6% dei casi | la sola terapia antibiotica orale risulta sicura ed efficace in alcuni casi di infezioni osteoarticolari, principalmente nei bambini di età inferiore ai 3 anni, poiché la Kingella kingae è l'agente patogeno più frequentemente coinvolto in questa gruppo.  E' raccomandata attenzione quando si sospetta infezione da Staphyloccoccu aureus, soprattutto nei pazienti ad alto rischio MRSA. In questo gruppo di bambini dovrebbe essere inizialmente somministrato un antibiotico per via endovenosa |
| McBride S 2018  USA | Osservazionale retrospettivo  coorte | 974 | Terapia antibiotica empirica ai giorni 0, 1 o 2 dall'ospedalizzazione  terapia antibiotica ai giorni di ospedalizzazione 0, 1 o 2 per MRSA (n=732) o MSSA (n=242)  gli antibiotici più frequentemente usati per la copertura MRSA: clindamicina, vancomicina, sulfametossazolo e trimetoprim e linezolid  gli antibiotici più frequentemente usati per la copertura MSSA: cefazolina, ceftriaxone, oxacillina e nafcillina  risultati colturali ai giorni 0, 1 o 2 dall'ospedalizzazione  confronto tra i due grupi di trattamento: riospedalizzazione, RMN rieseguita e miglioramento a 72 ore dei marker infiammatori | 1. rieseguita RMN entro 7 e 180 giorni: più frequente nei pazienti che hanno ricevuto una copertura antibiotica iniziale contro MRSA che nei pazienti che hanno ricevuto una copertura per solo MSSA (8,6% vs 4,1 % entro 7 giorni; 12% vs 5,8% entro 180 giorni)  2. la riospedalizzazione a 90 e 180 giorni è stata simile tra i due gruppi (9,0% vs 8,7% e 10,9% vs 11,2% rispettivamente)  3. pazienti con copertura antibiotica per MRSA e copertura antibiotica solo per MSSA hanno simili rate di miglioramento a 72 ore dei valori della proteina C reattiva, ma i pazienti con copertura contro MRSA hanno un più basso rate di normalizzazione dell'emocromo a 72 ore confrontato con i pazienti che hanno ricevuto una copertura antibiotica solo per MSSA (4,2% VS 16,4%) | un precoce trattamento antibiotico contro MRSA è associato a una più alto tasso di ripetizione della RMN se confrontato con pazienti che hanno ricevuto trattamento antibiotico per MSSA ma non per MRSA.  La riospedalizzazione è simile nei due gruppi. |
| Chiappini E  2018  Italia | Osservazionale  retrospettivo  coorte | 398 | cartelle cliniche di 19 centri pediatrici italiani di bambini con diagnosi di osteomielite acuta ematogena o osteomielite subacuta ematogena  i pediatri di ogni centro hanno compilato uno specifico questionario  Le variabili analizzate includevano: dati demografici, parametri clinici e di laboratorio, imaging, procedure chirurgiche, trattamento antibiotico, durata dell'ospedalizzazione, durata totale della terapia, complicanze e sequele | 1. Non ci sono state differenze tra bambini affetti da osteomielite acuta e subacuta ematogena eccetto che per la febbre all'esordio (63,0% vs 42,9%) e un coinvolgimento più frequente spinale nell'osteomieliti subacuta (6,7% vs 20,4%)  2. test microbiologici: batteri patogeni sono stati identificati in 93 bambini (23,4%). Sono stati isolati 58 ceppi di Staphylococcus aureus, 5 (8,6%) MRSA. Non è stato isolato in nessun caso Kingella kingae  3. Non sono stati evidenziati rischi differenti per complicanze/sequele nei due gruppi (osteomielite acuta 38,8% vs osteomielite subacuta 34,7%)  4. la durata e il tipo di terapia antibiotica non è associata a rischio di complicanze/sequele | Prevalenza di MRSA di circa l'8% nei bambini affetti da osteomielite acuta e subacuta ematogena |
| de Graaf  2017  Regno Unito | Osservazionale prospettico  coorte | 313 | sono stati valutati 44 centri secondari e terziari nel Regno unito  I casi in esame sono stati classificati come semplici (n=218) o complicati (infezioni da patogeni resistenti o virulenti, infezioni di un impianto o presenza di comorbidità)(n=95) | 1. I dati epidemiologi sono risultati conformi ai dati Europei  2. I bambini che hanno effettuato lo switch alla terapia orale entro 7 giorni dall'inizio della terapia endovenosa hanno avuto meno rischio di fallimento terapeutico (9,6%) rispetto ai bambini che hanno effettuato lo switch dopo i 7 giorni dall'inizio della terapia endovenosa (16,1% quando lo switch è stato effettuato tra 1 e 2 settimane; 18,2% quando è stato effettuato dopo 2 settimane) | un futuro trial randomizzato controllato sarebbe necessario per reclutare tutti gli ospedali terziari e la maggior parte dei secondari nel Regno Unito.  I clinici hanno implementato lo switch precoce alla terapia orale nei pazienti affetti da patologia non complicata senza evidenze basate su un trial clinico. Tuttavia i criteri per lo switch alla terapia orale non sono stati chiaramente definiti o basati su evidenze. |
| Chiappini  E  2017  Italia | Osservazionale retrospettivo  coorte | 121 | analisi retrospettiva dei dati raccolti in cartelle cliniche in un periodo di 6 anni  I dati demografici, dettagli clinici, risultati microbiologici e radiologici, trattamento antibiotico (tipo di farmaco e durata della terapia), necessità di una procedura chirurgica, riospedalizzazione nei 6 mesi dalla diagnosi iniziale sono stati inseriti in un database elettronico | 1. Febbre all'esordio: 55/121 (45,5%)  2. la sede più frequentemente coinvolta è l'arto inferiore 68/121 (56,2%)  3.la diagnosi microbiologica è stata ottenuta in 33,3% dei casi  4. lo Staphylococcus aureus è il patogeno più frequentemente isolato (n=20), MRSA n=0, PVL+ n=2  5. in 59/121 casi sono stati complicati. Nell'analisi univariata i fattori associati a osteomieliti acute ematogene complicate sono stati: recenti episodi febbrili , febbre all'esordio, coinvolgimento dell'arto superiore, conta dei globuli bianchi ≥ 12000 /microL, proteina C reattiva ≥ 10 mg/L, infezione da S.aureus. L'unico fattore di rischio per osteomielite complicate nell'analisi multivariate è lo S. aureus | Le complicanze sono frequenti nei bambini affetti da osteomielite acuta ematogena e sono frequentemente correlate all'infezione da parte dello S.aureus.  In questo studio la diagnosi microbiologica è stata ottenuta in un terzo dei casi.  Il trattamento empirico per lo Staphylococcus aureus meticillino sensibile sembra giustificato dai dati microbiologici |
| Patel L  2017  USA | Osservazionale  retrospettivo  coorte | 1602 | i pazienti sono stati selezionati retrospettivamente sulla base dell'imaging e test di laboratorio  I risultati clinici sono satati valutati considerando la degenza ospedaliera, il tempo della RMN, il tempo della somministrazione di antibiotici, il tasso di ricovero ospedaliero, il tasso di riosepdalizzazione a 30 giorni  le valutazione delle infezioni muscoloscheletriche nel dipardimento di emergenze sono state riviste da 1 anno prima dell'attuazione(n=383) a 2 anni dopo l'implementazione (n=1219) del CPM | 1.Un significativo miglioramento del tempo di somministrazione degli antibiotici dopo l'implementazione del CPM per tutti i pazienti (4,3 vs 3,7 ore) e per i pazienti con infezione muscoloscheletrica confermata (9,5 vs 4,9 ore)  2. il miglioramento del tempo complessivo per la RMN (13,2 vs 10,3 ore) e la degenza ospedaliera (4,7 vs 3,7 giorni), ma non è statisticamente significativo  3. il tasso di somministrazione di antibiotici e la riospedalizzazione a 30 giorni sono simili prima e dopo l'implementazione del CPM | l'implementazione del CPM nelle infezioni muscoloscheletriche ha standadizzato l'approccio alla valutazione e alla diagnosi delle infezioni muscoloscheletriche con conseguente diminuzione significativa del tempo di somministrazione degli antibiotici e una tendenza alla riduzione del tempo per la RMN e la degenza ospedaliera |
| Osei L  2017  Guyana Francese | Osservazionale  retrospettivo  coorte | 55 | raccolta di informazioni demografiche del paziente, presentazione clinica, decorso ospedaliero, reperti di laboratorio e microbiologici, studi radiologici, follow-up clinico | 1. nell'80% dei bambini con età ≥ 36 mesi hanno infezioni da Staphylococcus aureus meticillino sensibile in corso di infezioni cutanee non trattate  2. cinque bambini con infezioni multi-sistemica (fatale in 1 caso) principalmente dovuto a S.aureus PVL+  3. bambini di età compresa tra 6 e 36 mesi hanno più probabilmente infezioni coltura negativa, artrite settica e caratteristiche cliniche e biologiche più lievi | Studi prospettici futuri sono richiesti per guidare una diagnosi razionale e strategie terapeutiche |
| Benvenuti MA  2016  USA | Osservazionale  retrospettivo  coorte | 113 | i pazienti sono stati stratificati in due gruppi: infezione locale o infezione disseminata  dati demografici, risultati degli esami colturali, marker di severità (PCR, globuli bianchi, VES, parametri vitali), tempo di inizio della terapia antibiotica sono stati raccolti analizzando le cartelle cliniche | 1. in entrambi i gruppi (infezione locale e disseminata) pazienti con un esame tissutale colturale positivo sono significativamente più grandi di quelli con una coltura negativa  2. non sono state evidenziate differenze nelle coltura positive dopo la somministrazione della terapia antibiotica in entrambi i gruppi  3. non c'è stata alcuna significativa differenza nella durata del ricovero in entrambi i gruppi in base al tempo di somministrazione dell'antibiotico ( prima o dopo il tempo della coltura del tessuto)  4. S. aureus è il patogeno più frequentemente isolato, seguito dallo S.pyogenes  5. nel gruppo infezione locale, i pazienti che hanno ricevuto gli antibiotici successivamente rispetto al triage del pronto soccorso è stato significativamente meno probabile che venissero dimessi  6. pazienti con esame tissutale colturale negativo correlano una durata più breve del ricovero | lo studio dimostra che la somministrazione di antibiotici prima del esame colturale tissutale non correla con nessuna differenza nella sensitività colturale nei pazienti con infezioni muscoloscheletriche  la somministrazione precoce della terapia antibiotica è stato dimostrato essere correlata con una riduzione dell'ospedalizzazione nel gruppo a infezione locale |
| Chou AC  2016  Singapore | Osservazionale  retrospettivo  coorte | 24 | analizzate le cartelle cliniche di 24 pazienti pediatrici trattati con la sola terapia farmacologica (n=3) o in combinazione con la terapia chirurgica (n=21)  il trattamento medico prevede l'utilizzo di antibiotici (clozacillina, penicillina, amoxicillina/clavulanato, trimetroprim/sulfametossazolo, clindamicina, ceftriaxone, ampicillina) e supporto medico.  La chirurgia è indicata quando la dimensione maggiore della raccolta fluida è > 1 cm | 1. esame colturale positivo nell'88% dei pazienti. Lo S.aureus è stato isolato in 18 bambini; Salmonella gruppo-D in 3 bambini; nessun patogeno in 3 bambini  2. il tempo mediano dal ricovero alla diagnosi è stato 1,87 giorni  3. nei bambini trattati chirurgicamente dalla diagnosi al trattamento chirurgico è stato 1,19 giorni e dal ricovero alla chirurgia 2,86 giorni  3. quattro pazienti hanno effettuato terapia chirurgiche multiple, 2 dei quali hanno sviluppato un osteomielite cronica e sono stati coltura negativa e ritardo della chirurgia per più di 10 giorni | Precoci interventi chirurgici per le osteomieliti acute batteriche nei bambini aumentando la diagnosi colturale  In pazienti con raccolta fluida < 1cm della dimensione maggiore, il trattamento medico da solo è efficace  Pazienti con complicanze sono caratterizzati da coltura-negativa, multipli interventi chirurgici e chirurgia ritardata |
| Calvo C  2016  Spagna | Osservazionale  retrospettivo  coorte | 641 | analizzate la cartelle cliniche di bambini di età compresa tra 1 mese e 14 anni di 25 ospedali ed è stato compilato uno specifico questionario  Le variabili analizzate includono: dati demografici, clinica e parametri di laboratorio alla presentazione, trattamento antibiotico, durata dell'ospedalizzazione, complicanze, durata totale della terapia e sequele  la conferma di osteomielite e artrite settica richiede l'isolamento batterico positivo | 1.osotemielite n=298 (46%)  artrite settica n= 111 (36%)  osteoartrite n=78 (12%)  spondilodisciti n=33 (5%)  2. bambini con osteomielite sono più grandi dei bambini con artrite settica (63 mesi vs 43 mesi)  3. RMN e scintigrafia ossea rendimento per la diagnosi di osteomielite (94%)  4. artrocentesi è stata effettuata neò 96% dei casi di artrite settica  5. un microrganismo è stato isolato in 246 pazienti (38% dei casi; 33% osteomieliti vs 55% artrite settica): lo S.aureus è risultato essere il più frequente batterio isolato (63%). seguito da K.kingae (15%), S.pyogenes (9%).  6. il 95% dei bambini hanno ricevuto una terapia endovenosa iniziale : la maggior parte cefotaxime+cloxacillina (60%), o cloxacillina (40%)  7. la durata totale del trattamento è stata 38 (± 31) giorni per l'osteomielite; 28 (±16) giorni per l'artrite settica  8. il 20% dei bambini con osteomielite (46% per complicanze) e 53% dei casi delle artriti settiche (95% artrotomia iniziale) sono stati sottoposti alla chirurgia  9. non sono state osservate differenze cliniche confrontando i pazienti con artrite settica con iniziale artrotomia (n=123) vs artrocentesi (n=109), tranne nel più alto tasso di artrite settica dell'anca nel pazienti con iniziale artrotomia  10. bambini con artrocentesi hanno avuto meno sequele (6,6% vs 1%), ma non nell'analisi multivariata | S.aureus è il patogeno più frequentemente isolato e la K.kingae p stata rilevata in un'alta percentuale di casi  Il trattamento conservativo è stato effettuato nella metà dei pazienti  C'è un basso tasso di sequele, anche nel caso di un approccio non chirurgico |
| Roul-Levy A  2016  Francia | Osservazionale  retrospettivo caso-controllo  coorte | 45 | 19/45 bambini trattati esclusivamente in regime ambulatoriale per via orale (amoxicillina-clavulanato 80 mg/kg/die)  26/45 bambini hanno ricevuto un trattamento endovenoso di 2-4 giorni (cefamandole 150 mg/kg/die) prima dello switch alla terapia orale | non sono state osservate differenze significative tra i due gruppi di trattamento:  - il fallimento del trattamento è stato osservato in 3 pazienti nel gruppo a terapia endovenosa, vs 1 paziente nel gruppo a esclusiva terapia orale  - complicanze radiografiche : 1/26 vs 1/19  - complicanze generali: 2/26 vs 0/19  - durata del trattamento : IV 42,5 (34,5-51,5) vs PO 43 (28,5-46) | il trattamento ambulatoriale orale con amoxicillina-clavulanato sembra essere una valida alternativa alla classica terapia endovenosa e orale nel trattamento dell'osteomielite acuta ematogena in bambini senza gravi criteri |
| Ratnayake K  2015  USA | Osservazionale  retrospettivo  coorte | 67 | sono state analizzate le cartelle mediche di bambini presentati al pronto soccorso con diagnosi di osteomielite.  Sono stati analizzate gli esami colturali, l'emocolture e gli esami di imaging.  Sono stati identificati gli organismi patogeni, le ossa coinvolte, il tempo di trattamento antibiotico e le complicanze locali e ematogene | 1. il patogeno isolato più frequentemente è stato lo S.aureus meticillino sensibile (26/55), seguito da lo S.aureus meticillino resistente (21/55) (sensibile alla vancomicina e al triemtoprim-sulfametossazolo; due resistenti alla clindamicina).  2. l'osso più frequentemente interessato è stato il femore (24/73).  3. in 46 soggetti (69%) ci sono state 75 complicanze locali  4. il patogeno più comunemente presente in caso di complicanze è stato MRSA (49%)  5. tre pazienti hanno avuto complicanze ematologiche: trombosi venosa profonda, emboli polmonari settici, endoftalmiti  6. i soggetti con complicanze hanno ricevuto un trattamento antibiotico più breve (6 giorni (IQR4-9 giorni) vs 8 giorni (IQR 6-22 giorni))  7. quando un esame colturale operativo è stato effettuato dopo l'inizio della terapia antibiotica è risultato positivo per un patogeno nel 84% dei casi | il trattamento dell'osteomielite pediatrica dovrebbe includere la copertura antibiotica verso MRSA  Più frequentemente nei pazienti pediatrici sono interessate le ossa lunghe  Le complicanze locali sono comuni, soprattutto in caso di infezione MRSA  Le complicanze ematogene possono includere la trombosi venosa profonda e può essere correlato al trattamento con un catetere venoso centrale  La resa dell'esame colturale operativo quando la terapia antibiotica è già stata iniziata è elevata, il trattamento antibiotico non deve essere quindi ritardato fino a che non si ottiene un esame colturale positivo |
| Keren R  2015  USA | Osservazionale retrospettivo  coorte | 2060 | confronto con uno studio retrospettivo di coorte il PICC (n=1055) con la terapia orale (n=1005) per il trattamento dell'osteomielite acuta | 1. bambini trattati con antibiotici per via orale non hanno avuto più fallimenti terapeutici rispetto a quelli trattati con antibiotici per via PICC  2. gruppo PICC: 158 (15,0%) hanno presentato una complicanza del PICC che ha richiesto una visita al pronto soccorso (n=96), una riospedalizzazione (n=38) o entrambe (n=24)  3. i tassi reazione avversa al farmaco sono stati leggermente maggiori nel gruppo PICC | i medici dovrebbero riconsiderare la pratica del trattamento endovenoso prolungato in bambini altrimenti sani con osteomielite qualora esista un trattamento orale alternativo |
| Street M  2015  Nuova Zelanda | Osservazionale retrospettivo  coorte | 813 | analisi retrospettiva utilizzando le cartelle cliniche di bambini con osteomielite acuta ematogena in 2 reparti ortopedici di due ospedali | 1. incidenza 1:4000 , in diminuzione nel corso dei 10 anni in studio  2. più frequente nel sesso maschile  3. Maori (incidenza 1:2500) e isolani del Pacifico (incidenza 1:2500) sono stati sovra rappresentati  4. la diagnosi è stata fatta clinicamente nel 27% dei casi, radiologicamente nel 66% e chirurgicamente nel 7% dei casi  5. l'agente patogeno più comunemente isolato è stato lo S.aureus, con tasso di MRSA basso (2%)  6. la durata media del trattamento antibiotico endovenoso e orale è stata 43,7 giorni ; la durata media del trattamento endovenoso è stato 22,3 giorni; la durata media del trattamento orale è stato 21,4 giorni  7. l'antibiotico più utilizzato è stato la flucloxacillina: somministrato come farmaco di prima scelta in 482 bambini (59%) e in 268 (33%) è stato l'unico antibiotico somministrato; il secondo antibiotico più utilizzato è stato l'amoxicillina-clavulanato: prima scelta in 252 bambini (31%) e come unico farmaco in 227 bambini (28%). Quando non è stato identificato alcun patogeno l'amoxicillina-clavulanato è stato il farmaco più utilizzato  8. un totale di 361 pazienti (44%) hanno richiesto l'intervento chirurgico  9.il tasso di recidiva è stato il 6,8% e di complicanze correlato al trattamento del 15,2% | In Nuova Zelanda c'è una bassa incidenza di MRSA; la flucloxacillina rimane una buona scelta per il trattamento empirico in questa popolazione  il tasso di ricadute e cronicizzazione delle osteomieliti è basso; questo potrebbe essere spiegato dalla lunga terapia antibiotica ; tuttavia potrebbe essere anche correlato all'aumento del trattamento correlato alle complicanze |
| Liu RW  2013  USA | Osservazionale retrospettivo  coorte | 95 | 78 bambini di un ospedale universitario, 17 bambini di un ospedale della contea  sono state analizzati i dati demografici del paziente (età, genere, comorbidità), la presentazione (durata dei sintomi, malattie precedenti, febbre al momento della presentazione, VES, PCR al momento dell'ammissione o al primo giorno), sito di infezione, risultati colturali, trattamento (chirurgia, durata della degenza , antibiotici per via endovenosa o orale dopo la dimissione, durata degli antibiotici), recidiva, complicazioni del catetere (rimozione accidentale, malfunzionamento, infezione) e la data dell'ultimo follow-up | 1. non ci sono state differenze significative tra i due ospedali nei dati di base (età, sesso, durata dei sintomi, precedenti malattie, febbre al ricovero, VES, PCR)  2.c'è stato un significativo più alt tasso di uso di antibiotico endovenoso alla dimissione nel gruppo universitario (95% vs 65%)  3. la durata della terapia endovenosa e totale è stata simile nei due ospedali eccetto in caso della durata totale del trattamento antibiotico in bambini alla dimissione con antibiotici per via orale (21 giorni nel gruppo universitario vs 44 giorni nell'altro gruppo)  4. ci sono state 8 recidive nell'ospedale universitario; 0 nell'ospedale della contea (non significative)  5. ci sono state 16 (2 complicanze maggiori) complicanze nel catetere nell'ospedale universitario; 1 (0 complicanze maggiori) nell'ospedale della contea (non significative).  Nell''ospedale universitario 2 su 16 pazienti con complicanze legate al catetere hanno sviluppato ricorrenze.  6. c'è una significativa durata più lunga del follow-up nell'ospedale della contea (32 mesi vs 6 mesi)  7. il gruppo endovenoso presenta una maggior durata dei sintomi (13,5 vs 5,2 giorni) e una tendenza ad una PCR iniziale più elevata (6,9 vs 3,5)  8. la maggior parte delle infezioni in entrambi gli ospedali è causata da S.aureus o coltura negativa  9. dei 43 pazienti trattai chirurgicamente 4 hanno avuto recidive (9%); dei pazienti non trattati chirurgicamente 4 dei 52 pazienti hanno avuto recidive(8%) | la transizione precoce alla terapia antibiotica orale può ha un tasso di recidiva simile alla terapia endovenosa , con un ridotto tasso di complicazioni legati all'utilizzo di cateteri venosi centrali |
| Arnold JC  2012  USA | Osservazionale retrospettivo  coorte | 194 | un ricerca di 8 anni della cartelle mediche di bambini con infezioni osteoarticolari  Sono studiati solo i bambini con artrite batterica acuta coltura positiva (ABA=32) o osteomielite acuta batterica (ABO=113) o entrambe (ABA+ABO=49)  E' stata eseguita un'analisi dei dati demografici, clinici, referti radiologici, test di laboratorio (risultati colturali, analisi del liquidi con artrocentesi, risultati colurali dei tessuti prelevati chirurgicamente) , dati relativi al ricovero, dati ambulatoriali, le complicanze e le sequele a lungo termine. | 1. Di 194 pazienti totali, ci sono state 40 complicanze delle quali 35 in caso di terapia prolungata. C'è stato solo 1 fallimento microbiologico, probabilmente per una ritenzione intra-articolare di un frammento di osso infetto.  2. la PCR è stata più alta all'inizio nei pazienti con simultaneo ABO+ABA e nei pazienti con complicanze. ed era più bassa al passaggio alla terapia orale nel gruppo con complicanze (1,5 vs 2,1 mg/dL)  3. il patogeno più frequentemente isolato è stato lo S.aureus meticillino sensibile (61%), seguito da S.pyogenes (13%), S.aureus meticillino-resistente (7,2%) e K.kingae (6,2%). MRSA è stato responsabile del 9% delle ABO, ma non ha causato nessun caso di ABA isolata; K. kingae è stata responsabile del 19% dei casi di ABA e del 3% dei casi di ABO  4. interventi chirurgici multipli sono stati effettuati più frequentemente nelle infezioni MRSA rispetto alle infezioni MSSA (8/ 14 (57%) vs 25/19 (21%)).  5. Le complicanze e le sequele tendono ad essere più frequenti nelle infezioni MRSA (5/14, 36%) rispetto che nelle MSSA (26/119, 22%).  6. Nove bambini con infezioni MRSA hanno ricevuto una terapia empirica con vancomicina e in 7 casi si è passato a clindamicina entro 8 giorni; i restanti 2 bambini sono stati trattati con vancomicina parenterale per più di 4 settimane; 1 bambino ha effettuato lo switch alla terapia orale con clindamicina, e 1 alla terapia orale con trimetoprim-sulfametossazolo | La combinazione di parametri clinici (risoluzione della febbre, riduzione del dolore, miglioramento funzionale ) e parametri di laboratorio (valori della proteina C reattiva vicini al valore normale ) è utile per lo switch alla terapia orale nei bambini con infezioni osteoarticolari.  Le complicanze sono associate a più alti valori di PCR alla diagnosi e più bassi valori alla fine della terapia parenterale, suggerendo che i clinici sono stati molto più conservativi con la terapia prolungata iniziale parenterale in questo gruppo. |
| Chen WL  2010  Taiwan | Osservazionale retrospettivo | 27 | analisi retrospettiva di bambini ricoverati in ospedale con diagnosi di infezioni osteoarticolari (n=27) e i risultati sono stati confrontati con le pubblicazioni dei passati 10 anni sulle infezioni osteoarticolari in Taiwan (n=692) | 1. dei 27 bambini 15 (55,6%) hanno avuto concomitante interessamento osseo e articolare  2. emocoltura positiva nel 44,4% dei casi dello studio e nel 48-52% negli altri studi  3. il patogeno è stato identificato nel 66,7 % dei bambini nello studio e nel 63-76% degli altri studi, quando sono stati disponibili campioni chirurgici  4. In tutti gli studi il patogeno più frequente è stato lo S. aureus, di questo nel 13,3% dei bambini in studio era meticillino resistente, nel 22-24% negli altri studi  5. tutti i bambini in studio hanno ricevuto antibiotici parenterale seguiti dalla terapia orale | Osteomieliti e artrite settica combinate sono presenti in più della metà dei pazienti.  Gli effetti delle infezioni combinate ossee e articolari nella crescita dell'osso devono essere ancora determinate  Interventi chirurgici rimangono un importante componente della gestione delle infezioni osteoarticolari |
| Jagodzinski NA  2009  Australia | Osservazionale prospettico  coorte | 70 | Studio bi-centrico  i pazienti hanno iniziato una terapia antibiotica parenterale ed è stato seguito un algoritmo di trattamento predeterminato (flucloxacillina)  Tutti i pazienti con artrite settica sono stati sottoposti a washout articolare  Il passaggio alla terapia orale è stato e stato effettuato una volta che si è registrato un miglioramento delle condizioni cliniche e dei parametri ematologici  E' stato eseguito un follow-up continuo per 1 anno con esami ematici e radiografie | 1. Stafilococchi sono stati gli unici patogeni isolati in caso di osteomielite; infezioni da Streptococco sono state molto più frequenti nei bambini con artrite settica  2. nel 59% dei bambini è stato possibile lo switch alla terapia orale dopo 3 giorni di terapia parenterale; 86% dopo 5 giorni.  3. La durata mediana della terapia durante l'ospedalizzazione è stata 5 giorni | La terapia antibiotica a breve durata parenterale e orale è efficace nel trattamento dei bambini con infezioni acuta osteoarticolare non complicata |
| Spyridis N  2016  Belgio | Osservazionale  retrospettivo  survay | 84 ospedali | E' stato compilato un questionario online da parte degli ospedali partecipanti sulla disponibilità e le caratteristiche delle linee guida di prescrizione degli antibiotici e sul trattamento empirico incluso la durata della terapia per le 5 sindromi infettive più comuni: tratto respiratorio, urinario, cute e tessuti molli, osteoarticolari e la sepsi nei neonati e nei bambini | 1.84 ospedali da 19 paesi Europei hanno partecipato al sondaggio  2.74 hanno confermato l'esistenza di linee guida  3.Linee guida complete sono state riportate nel 20% degli ospedali e la maggior parte utilizza un range di differenti fonti (71%).  4. Penicillina e amoxicillina sono stati i farmaci più frequentemente raccomandati per le infezioni del tratto respiratorio (fino al 76); le cefalosporine per le infezioni del tratto urinario (fino al 50%) e per le infezioni della cute e dei tessuti molli (20%) e delle ossa (30%).  5. La terapia antibiotica per infezioni ossee e cutanee è solitamente empirica poiché è raro isolare il patogeno nei bambini. Molti ospedali hanno raccomandato peniciline antistafiloccociche per le infezioni della cute e tessuti molli (43%) e ossee (36%) aventi come target lo S.pyogenes e lo S.aureus meticillino -sensibile. Soltanto un piccolo numero di ospedali ha raccomandato di usare glicopeptidi (vancomicina) o lincosamidi (clindamicina) come terapia empirica nelle infezioni osteoarticolari nonostante le recenti raccomandazioni di utilizzare questi antibiotici solo se il tasso di MRSA sia superiore al 10%  6. raccomandazioni per la sepsi neonatale includono 20 differenti combinazioni di antibiotici  7. la durata della terapia secondo le linee guida è stata disponibile soprattutto per le infezioni del tratto respiratorio e urinario (82%). Un terzo degli ospedali con linee guida ha fornito raccomandazioni sulla durata della terapia nella sepsi  Le linee guida comunemente disponibili sono state quelle delle infezioni del tratto urinario (74%), sepsi neonatale (71%) e sepsi nei bambini (65%) | Le raccomandazioni sulla terapia antibiotica in Europa sono generalmente carenti negli ospedali pediatrici  Sono stati documenti numerosi antibiotici e diverse combinazioni di antibiotici per la maggior parte delle infezioni  E' richiesto quindi un miglioramento delle linee guida e della loro evidence based che colleghi la terapia empirica ai tassi di resistenza antibiotica nei vari paesi |
| Sánchez-Moreno P  2015  Spagna | Osservazionale retrospettivo  coorte | 117 | studio retrospettivo in bambini con infezioni osteoarticolari in un ospedale terziario | 1. I casi analizzati sono stati 117: 78 casi artrite settica SA (66,6%), 25 casi osteomielite OM (21,3%), 14 casi osteoartrite OA (11,9%)  2. SA più frequente nelle femmine 54%, OM nei maschi 60%  3. età mediana alla diagnosi maggiore nelle OM rispetto alle SA (47 vs 31 mesi)  4. La febbre è meno comune nelle SA che nelle OM (79% vs 84%)  5. il dolore è il sintomo più frequente nelle OM (68%) e il gonfiore nelle SA (41%)  6. precedenti fattori di rischio sono stati riscontrati più frequentemente nelle SA (51% vs 26%)  7. il ginocchio è stata la sede più comune nelle SA (59%), mentre il femore e il tarso (32%) nelle OM.  8. il valore mediano al momento dell'ammissione della PCR è stato 49,7 mg/L nelle SA e 56,2 mg/L nelle OM  9. il valore iniziale della VES non era comune (13%)  10. convenzionalmente sono state effettuate indagini radiografiche con bassa resa per la diagnosi di entrambe le patologie  11. l'ecografia è risultata compatibile nel 92% dei casi delle SA e nel 75% dei casi delle OM.  12. la RMN e la scintigrafia ossea hanno avuto la massima resa per la diagnosi di entrambe (100%). La RMN è stata effettuata nell'88% dei casi di OM vs il 15% dei casi di SA; la scintigrafia ossea è stata effettuata nell'o'88% dei casi di OM vs 7% dei casi di SA.  13. l'artrocentesi è stata eseguita nel 77% dei casi di SA, il liquido articolare è stato ottenuto nel 73% dei casi e le colture sono risultate positive nel 24,5% dei casi.  14. l'emocoltura è stata effettuata nel 71% dei pazienti (36% positivi in caso di SA, 32% positiva in caso di OM).  15. Lo S.aureus è stato il patogeno più frequentemente isolato nel liquido articolare e nel sangue  16. molti pazienti hanno ricevuto un trattamento antibiotico con cefotaxime e cloxacillina, per una durata mediana di 10,45 (±5) giorni per le OM e 7,7 (±4,5) giorni per le SA.  17. Cefadroxile è stato l'antibiotico orale più frequentemente somministrato (8%%).  18. La durata totale del trattamento è stata 27 (±16) giorni nelle OM e 26,1 (±16) giorni nelle SA  19. il 16% dei bambini con OM e il 27% dei bambini con SA hanno effettuato un intervento chirurgico  20. solo il 5% dei bambini con SA hanno avuto complicanze o sequele | le caratteristiche dei pazienti nello studio sono simili ai dati riportati in letteratura |
| Ferroni A  2012  Francia | Osservazionale prospettico  coorte | 197 | studio prospettico per analizzare l'epidemiologia e l'evoluzione clinica delle infezioni osteoarticolari al fine di validare l'adeguatezza del trattamento possibile di prima linea (cefamandl+gentamicina)  Tutti i bambini con sospetto di infezioni osteoarticolari acquisiti in comunità sono stati inclusi ed è stato effettuato un follow-up per 3 anni  la diagnosi eziologica è fatta in base all'emocoltura, l'aspirato articolare e punture ossee  Effettuata PCR due volte a settimana | 1. sono stati inclusi nello studio 197 bambini: 98 con artrite settica , 23 con osteoartrite settica, 70 con osteomielite e 6 con spondilodisciti  2. nel 44% dei casi si è sospettata una porta d'ingresso, tra cui il 55% di infezioni otorinolaringoiatriche  3. la proteina C reattiva è stato il marker infiammatorio più sensibile  4. La PCR ha aumentato del 54% le prestazioni della diagnosi batteriologica  5. tra i pazienti completamenti studiati (emocolture e campioni osteoarticolari), il 63% ha documentato un'infezione osteoarticolare.  6. i principali patogeni isolati sono stati K.kingae (52%), S.aureus (28%), S.pyogenes (7%), S.pneumoniae (3%) e S.agalactiae (2%)  7. tutti batteri isolati sono risultati sensibili in vitro al trattamento probabilistico e l'esito è stato favorevole | la PCR ha migliorato significativamente le prestazioni e il ritardo di diagnosi in caso di infezione osteoarticolare nei bambini, per il quale la K.kingae si è rivelato essere il primo agente eziologico.  Il trattamento probabilistico è stato attivo nei confronti dei principali batteri responsabili delle infezioni osteoarticolari in età pediatrica  Questo protocollo può essere attivato solo nelle regioni a bassa prevalenza di MRSA poiché non sono stati isolati nello studio batteri MRSA |
| Lakshminarayana I.  2012  Regno Unito | Osservazionale retrospettivo  coorte | 12 | sono stati analizzati retrospettivamente le notifiche di bambini con osteomielite durante un periodo di 2 anni | 1. la temperatura ≥ 38 gradi era presente soltanto in 1/3 dei bambini; il dolore e la riduzione funzionale erano presenti in tutti i bambini  2. un'infezione recente o un trauma erano presenti nella maggior parte dei bambini  3. sono state rilevate VES elevata (> 35 in 10 pazienti) e alta conta piastrinica (> 400 in 11 pazienti)  4. la proteina C reattiva è risultata < 15 in 3 pazienti  5. le emocolture sono risultate negative in 11 casi; l'unico caso positivo è risultato dovuto a Streptococco gruppo B isolato in un neonato da sangue e su aspirazione articolare  6. la RMN è stata utile per la conferma diagnostica nei casi dubbi  7. la scintigrafia ossea è stata eseguita in 4 casi per la diagnosi  8. Flucloxacillina è stato l'antibiotico più efficace nella maggior parte dei casi eccetto che nell'osteomielite del neonato  9. sei bambini hanno ricevuto la terapia antibiotica per 42 giorni, 1 bambino per 5 giorni e 1 bambino per 70 giorni, 4 bambini per 14 giorni  10. la durata della terapia antibiotica parenterale è stata molto variabile ed è stata guidata dalla risoluzione dei marker infiammatori  11. gli antibiotici sono stati iniziati a partire dal 12 giorni per un bambino  12. tre bambini hanno avuto complicanze, 1 bambino ha richiesto una sequestrectomia, uno ha sviluppato un ascesso di Brodie e uno ha sviluppato un'osteomielite multifocale cronica | osteomielite acuta è ancora una patologia a difficile diagnosi, sono perciò necessari un alto indice di sospetto insieme a segni clinici, test radiologici e di laboratorio per effettuare una diagnosi precoce ed iniziare il trattamento per evitare le complicanze a lungo termine |
| Nathawad R  2012  USA | Osservazionale retrospettivo  coorte | 46 | è stato effettuata una analisi retrospettiva in bambini ammessi in ospedale con diagnosi di osteomielite o artrite settica  sono stati valutati i dati demografici, i sintomi, diagnosi, tipo di patogeni isolati in coltura, marker infiammatori, imaging, trattamento ospedaliero, trattamento ambulatoriale e la durata totale della terapia | 1. dei 46 pazienti studiati: 13 con SA, 37 con OM, 4 con SA+OM  2. una diagnosi microbiologica è stata effettuata in 76,6% dei bambini con OM e nel 100% dei bambini con SA. Il patogeno più frequentemente isolato è stato lo S.aureus responsabile di 22 casi (48%).  3. i pazienti sono stati inizialmente trattati in regime di ricovero con la somministrazione di un antibiotico endovenoso (inizialmente empirico, poi modificato sulla base dei risultati colturali) e dopo la dimissione sono stati trattati a casa per via endovenosa con un catetere centrale (46%) e con la terapia orale (54%) con un antibiotico beta-lattamico (dicloxacillina, amoxicillina/clavulanato, penicillina VK, cefalexina), clindamicina, trimetoprim-sulfametossazolo e fluorochinoloni. La terapia a casa è stata ben tollerata ed efficace in tutti i casi  4. i pazienti con osteomielite acuta sono stati trattati per una durata tra i 28 e 63 giorni, eccetto per 1 paziente con infezione da Mycobacterium tuberculosis che è stato trattato per 1 anno | i risultati hanno dimostrato che personalizzare un efficace regime terapeutico domiciliare è efficace ma richiede una specifica diagnosi microbiologica |
| Zaoutis T  2009  USA | Osservazionale retrospettivo  coorte | 1969 | è stato condotto uno studio retrospettivo di coorte su bambini con diagnosi di osteomielite acuta per confermare il cambiamento nell'assunzione precoce della terapia orale  sono stati divisi i bambini in due gruppi: terapia endovenosa prolungata e precoce switch alla terapia orale | 1. dei 1969 pazienti selezionati, 1021 hanno ricevuto una terapia prolungata endovenosa, 948 hanno ricevuto una terapia orale. L'uso di una terapia venosa prolungata varia significativamente negli ospedali (10-95%)  2. il tasso di fallimento del trattamento è stato del 5% (54/1021) nei gruppo con terapia endovenosa prolungata e del 4% (38/984) nel gruppo di terapia orale. Non c'è stata un'associazione significativa tra fallimento terapeutico e modalità della terapia antibiotica  3. il tempo mediano di fallimento terapeutico è stato simile nei due gruppi (16,5 giorni nel gruppo terapia endovenosa prolungata vs 14,0 giorni nel gruppo terapia orale)  4. nel gruppo della terapia prolungata endovenosa 35 bambini (3,4%) sono stati riospedalizzati per una complicanza legata al catetere | il trattamento dell'osteomielite acuta con switch precoce alla terapia orale non è associato a un rischio più elevato di fallimento terapeutico ed evitare i rischi legati al trattamento endovenoso prolungato |
| McNeil JC  2017  USA | Osservazionale retrospettivo | 192 | i casi sono stati selezionati da un database del servizio di malattie infettive ospedaliere | 1. i pazienti dimessi con antibiotici orali hanno avuto una durata più breve della febbre, un declino più rapido della proteina C reattiva e meno probabilmente erano affetti da MRSA  2. la frequenza delle complicanze ortopediche non è aumentata nei pazienti che hanno ricevuto una transizione preoce alla terapia antibiotica orale  3. per i pazienti con batteriemia da MRSA, le percentuali di complicanze tra coloro che hanno ricevuto ≥ 7 giorni rispetto a < 7 giorni di vancomicina non erano differenti  4. valori sierici di vancomicina > 15 microg/ml non erano associati ad una diminuzione della durata della febbre, batteriemia o ospedalizzazione, necessità di ripetere l'operazione o complicanze ortopediche, ma erano meno associati ad AKI | Le infezioni osteoarticolari da S.aureus sono associate a una morbilità sostanziale  cicli prolungati di vancomicina e valori sierici di vancomicina > 15 microg/ml non erano associati a esiti migliori per infezioni da MRSA |
| Brady PW  2014  USA | osservazionale | 31 | sono stati inclusi in un intervento multiforme (strumento decisionale condiviso, istruzione per docenti e tirocinanti) 12 ospedali universitari e 53 studi medici per indirizzare chi si prende cura dei bambini con osteomielite in tempo quasi reale e rafforzare le raccomandazioni basate sull'evidenza | la percentuale di bambini con osteomielite dimessa in terapia orale è aumentata da una mediana dello 0% al 100% entro 1 mese ed è stata mantenuta per oltre 1 anno | anche per le condizioni non comuni, l'adozione di prove rapide e sostenute è possibile usando i metodo di miglioramento della qualità |
| Giordano M  2019  Italia | Osservazionale retrospettivo | 150 | studio retrospettivo che include pazienti affetti da infezioni muscoloscheletriche  i dati sono stati ottenuti dalle cartelle cliniche  sono stati valutati dati demografici dei pazienti, sito di infezione, febbre e durata dei sintomi, storia di trauma precedente, durata dell'ospedalizzazione e valutazione cliniche , dati di laboratorio, dati microbiologici, imaging.  I pazienti sono stati classificati in 3 gruppi in base all'età:  gruppo A: pazienti dalla nascita-1 anno  gruppo B: 1 anno-4 anni  gruppo C: 4 anni-17 anni  il protocollo diagnostico e terapeutico: terapia endovenosa parenterale durante il periodo diagnostico seguito dallo switch alla terapia orale in accordo al tipo di patogeno isolato e ai sintomi sistemici | 1. soltanto il 31% dei patogeni è stato identificato. Il patogeno più comune è stato lo S.aureus meticillino-sensibile ; è stato osservato un aumento dei casi di infezioni da aperte di K.kingae e S.aureus meticillino-resistent  2. la durata mediana della terapia antibiotica è stata 6,8 settimane  3. è stata osservata una correlazione significativa tra l'età e i livelli sierici di proteina C reattiva | Aumento dell'incidenza delle infezioni muscoloscheletriche nel corso degli anni  Tra gli altri patogeni MRSA ha mostrato un alto tasso di coinvolgimento delle fisi |
| Merali HS  2014  USA | Review sistemica |  | Review sistemica usando PubMed.  Lo scopo di questa review è stato determinare se l’approccio empirico del trattamento antibiotico per le osteomieliti acute fosse efficace e sicuro confrontato con la terapia mirata. | - | Gli autori non sono riusciti ad individuare studi sull’efficacia e sulla sicurezza e raccomandano di effettuare uno studio randomizzato controllato per valutare il trattamento antibiotico ottimale |
| Grimbly C  2013 | Review sistemica |  | Creare un protocollo per una review sistematica basata sulle linee guida PRISMA al fine di valutare la letteratura, cercando evidenze sulla durata ottimale della terapia parenterale e orale. | È stata effettuata una review sistemica di 3400 studi confrontata con i 284 dello studio di Le Saux et al. | Questa review permetterà di fornire un’analisi statistica sulla terapia parenterale breve o prolungata valutando quale dei due regimi terapeutici fornisce il miglior outcome clinico |
| Howard-Jones AR  2013 | Review sistemica |  | Review sistemica della letteratura sulle osteomieliti acute nei bambini per determinare se la durata breve della terapia antibiotica confrontato con un trattamento prolungato hanno differenti percentuali di guarigione.  Sono stati anche analizzati gli studi per confrontare i diversi tassi di successo dei diversi antibiotici.  Sono stati inclusi trial controllati randomizzati, studi di coorte, studi caso-controllo e serie di casi | Sono stati selezionati 6 trial randomizzati controllati:   - 3 sulla durata della terapia antibiotica - 3 sul tipo di antibiotico usato   per il trattamento dell’osteomielite acuta ematogena | La qualità delle prove sul trattamento antibiotico dell’osteomielite acuta ematogena è limitata, permettendo solo raccomandazioni deboli (2B).  Questa review suggerisce che lo switch precoce dalla terapia ev alla terapia per os dopo 3-4 giorni nei pazienti che rispondono bene alla terapia, seguita da un periodo di 3 settimana di terapia orale è efficace come la terapia prolungata nei casi di osteomielite acuta non complicata. |
